# Herpes Simplex Virus, Alzheimer’s Disease and a Possible Role for Rab GTPases

**DOI:** 10.3389/fcell.2019.00134

**Published:** 2019-08-07

**Authors:** Elaine L. Bearer, Chengbiao Wu

**Affiliations:** ^1^Department of Pathology, University of New Mexico Health Sciences Center, Albuquerque, NM, United States; ^2^Department of Neurosciences, University of California, San Diego, La Jolla, CA, United States

**Keywords:** axonal transport, HSV (herpes simplex virus), Rab GTPases, squid giant axon, endosomes, dynein, kinesin, Alzheimer’s disease

## Abstract

Herpes simplex virus (HSV) is a common pathogen, infecting 85% of adults in the United States. After reaching the nucleus of the long-lived neuron, HSV may enter latency to persist throughout the life span. Re-activation of latent herpesviruses is associated with progressive cognitive impairment and Alzheimer’s disease (AD). As an enveloped DNA virus, HSV exploits cellular membrane systems for its life cycle, and thereby comes in contact with the Rab family of GTPases, master regulators of intracellular membrane dynamics. Knock-down and overexpression of specific Rabs reduce HSV production. Disheveled membrane compartments could lead to AD because membrane sorting and trafficking are crucial for synaptic vesicle formation, neuronal survival signaling and Abeta production. Amyloid precursor protein (APP), a transmembrane glycoprotein, is the parent of Abeta, the major component of senile plaques in AD. Up-regulation of APP expression due to HSV is significant since excess APP interferes with Rab5 endocytic trafficking in neurons. Here, we show that purified PC12-cell endosomes transport both anterograde and retrograde when injected into the squid giant axon at rates similar to isolated HSV. Intracellular HSV co-fractionates with these endosomes, contains APP, Rab5 and TrkA, and displays a second membrane. HSV infected PC12 cells up-regulate APP expression. Whether interference with Rabs has a specific effect on HSV or indirectly affects membrane compartment dynamics co-opted by virus needs further study. Ultimately Rabs, their effectors or their membrane-binding partners may serve as handles to reduce the impact of viral re-activation on cognitive function, or even as more general-purpose anti-microbial therapies.

## Introduction

Herpes simplex virus Type 1 (HSV) is a human enveloped double-stranded DNA virus and member of the alpha herpesvirus subfamily of *Herpesvirdiae*. HSV is prevalent in virtually all populations worldwide, with an estimated 85% infection rate in adults in the United States. HSV primarily enters epithelial cells of mucous membranes and replicates within them, then secondarily enters adjacent sensory nerve termini. Once inside the neuron, HSV travels retrograde within the sensory process to reach the neuronal cell nucleus in the trigeminal ganglion, which lies just outside the brain stem, where virus either replicates or goes latent. Latent viral DNA may re-activate in human, expressing viral proteins to produce new infective virus. Newly produced virions then travel outward by anterograde transport (Holland et al., 1999) from the neuronal cell body, a process referred to as egress, to either re-infect the mucous membrane, or to enter the brain (Bearer, 2004).

Herpes simplex virus is an enveloped virus. Thus, most steps in its life cycle depend on membrane trafficking within infected cells. Hence the Rab proteins, small GTPases, which are master regulators of cellular membrane dynamics (Schwartz et al., 2007; Zhen and Stenmark, 2015; Pfeffer, 2017), are thought to play intimate roles in many stages of HSV infection, transport, envelopment and egress (Raza et al., 2018; Spearman, 2018). Emerging exciting evidence links HSV re-activation in the brain with Alzheimer’s disease (AD; Itzhaki et al., 2016). This short commentary will integrate prior knowledge, provide new information and perspective, and conclude with a discussion about how Rab GTPases may serve as targets for HSV-AD interventions.

## HSV Infection and Alzheimer’s Disease

New observations have sparked renewed investigation into the possibility that HSV enters the brain, causing pathology. Two of these are particularly intriguing: (1) HSV interacts dynamically with host cell amyloid precursor protein (APP; Satpute-Krishnan et al., 2003; Cheng et al., 2011). APP is the parent protein that when cleaved and aggregated produces the Abeta of amyloid plaques of AD. (2) Trigeminal neurons also project into the brain stem (Bearer, 2004).

The idea for a link between HSV and AD was first substantiated in a small (*n* = 14) cohort of autopsied AD and normal brains tested for HSV DNA in three brain regions by PCR (Jamieson et al., 1991). Thereafter much controversy arose when another study did not identify HSV in post-mortem brains from subjects with AD (Hemling et al., 2003). HSV DNA was also reported by both PCR and *in situ* hybridization in cells in the temporal lobe of three patients with familial AD, with signal present in the cytoplasm, evidence of viral replication (Mori et al., 2004). The conflicting results from these may be due to sampling errors, with few individuals and small amounts of brain from selected areas tested.

Subsequently further support for the idea that a chronic low level of viral replication occurs and may damage the brain came from another study examining post-mortem brains for acute phase antibodies to HSV proteins (Wozniak et al., 2005). HSV re-activation in brain was proven in a study from an immuno-competent child infected at birth who underwent a brain biopsy for diagnosis of a cerebral mass found incidentally by radiology after a minor head injury at 8 years of age (Brown et al., 2010). HSV 2 was specifically identified by PCR from DNA in the biopsy. Immunohistochemistry for viral proteins detected intracellular virions. Abeta fibrils which sometimes aggregated into senile-plaque-like structures were detected by silver staining in the biopsy (Bearer, 2012). Follow-up for 6 years demonstrated on-going viral replication that ultimately resulted in behavioral changes, developmental delay and attention deficit. On-going viral replication in infants treated initially with acyclovir is now recognized by pediatricians and new long-term treatment protocols developed.

No study has yet tested the trigeminal nucleus (TGN) in the brain stem for HSV, which would be a likely portal for HSV entry into the brain from a lip infection (Bearer, 2004), nor the adjacent locus coeruleus (LC), where AD pathology may begin (Braak et al., 2011). Only one of the adult post-mortem studies, Mori et al., was designed to determine whether the distribution of HSV infection correlated with AD pathology anatomically. Interestingly, the TGN is anatomically adjacent to LC in the human brain, and TGN sends projections directly to the LC. Thus all of these reports would easily under-estimate the incidence, prevalence and amount of HSV in human brain.

A challenge to correlating anatomical location of HSV DNA with plaques and tangles is the fact that HSV travels long distances inside neuronal projections after replication in the cell body. Hence the location of viral replication, where HSV DNA is most abundant and thus detectable by PCR, may not be the site of plaque pathology, which appears to occur primarily at distant synapses.

Despite these challenges and the early conflicting results, evidence continues to accumulate linking HSV in the brain as a risk factor for onset and/or progression of AD in vulnerable people (Itzhaki et al., 2016). One of the genetic vulnerabilities identified in HSV-AD correlations is inheritance of the ApoE-*epsilon*4 allele (Itzhaki and Wozniak, 2006). ApoE-*epsilon*4 variant is the greatest known genetic risk factor for late-onset sporadic AD in a variety of ethnic groups (Lovheim et al., 2019), although 30% of people with AD do not have this allele. However, both HSV infection and ApoE4 alleles are common, hence some overlap is expected. The Arizona ApoE cohort, based in the Mayo clinic, is attempting to address this question through a longitudinal clinical study (Caselli et al., 2009).

The idea that HSV re-activation is correlated with progressive cognitive impairment has found significant support from large studies measuring anti-HSV antibodies in serum as a measure of viral activity (Letenneur et al., 2008; Lovheim et al., 2019). Each of these studies followed antibody titers in serum and cognition in large cohorts of cognitively normal elderly adults for many years and found strong statistical evidence of decreasing cognition with episodes of elevated anti-HSV titers. Additional evidence includes presence of HSV DNA in Abeta plaques in human post-mortem brains (Wozniak et al., 2009); up-regulation of APP expression in HSV infected cells (Cheng et al., 2011); accumulation of p-tau in HSV-infected Vero cells that also deposit Abeta and express secretase (Wozniak et al., 2007); and interference by HSV infection with amyloid processing (Shipley et al., 2005). However, to date Koch’s postulates, long the gold standard for establishing pathogen-disease causality, have not been satisfied (Bearer, 2012).

Alternative approaches to address the question of causal links between HSV and AD have turned up new information and raised new hypotheses of relationships. A series of publications assert that Abeta may be part of the innate immune system, serving to sequester, and perhaps kill, bacteria in the brain. In this scenario, HSV’s up-regulation of Abeta would be protective, although the excess Abeta in the end would be neurotoxic and damaging (Kumar et al., 2016; Moir et al., 2018). When HSV infective particles are injected into the mouse brain, APP is rapidly produced and after 21 days plaques form in surviving mice (Eimer et al., 2018). HSV is a human virus and is rapidly fatal in mice, with the latency seen in human infections apparently occurring less frequently in mouse.

Another study, designed to map and compare biological networks underlying two distinct AD-associated phenotypes, used multiple independent datasets collected from human subjects (Readhead et al., 2018). This study compared post-mortem brains that met neuro-pathological criteria for AD from individuals who were cognitively intact at the time of death, and compared their multi-omic network with brains from cognitively impaired individuals. Viral activity was evaluated in a multi-scale network analysis of four large, multi-omic datasets enabling direct examination of viral DNA and RNA sequences and host genome. This functional analysis of network patterns revealed roles for viral mediators in AD. An increased abundance of human herpesvirus HHV-6A DNA was found in AD; HHV-7 and both HSV 1 and 2 were also identified. Increased abundance of viral RNA, an indication of gene activity, was associated with cognitive impairment. Addressing one of the most pressing questions – Why, when so many are infected, do so few fall to AD? – this study also looked for quantitative trait loci that conferred increased risk of AD with viral load and viral gene expression. These findings were reproduced in studies of other cohorts in which increased HHV-6A and HHV7 as well as increased abundance of the HSV-1 latency-associated transcript (LAT) were found. These findings support the idea that multiple viruses contribute to AD, with strong contribution from the *Roseola viridae*, HHV-6A and HHV-7, as well as HSV. The findings that the roseola subfamily of *Herpesviridae* is involved in AD raise important public health issues, since these viruses are commonly contracted in early childhood, incidence may be higher in children living in poverty, and once infected, the virus endures throughout life in the brain. Currently roseola is considered a nuisance – there are no vaccines or other preventions available, and treatment of the acute infection is supportive.

Encouraging results from the Valacyclovir Clinical Trial for Mild Cognitive Impairment, hosted by the National Institute on Aging^1^ show that a treatment to block herpesvirus replication in mild cognitive impairment has benefit, and is thus not a far-fetched notion. Valacyclovir, a form of acyclovir, the first anti-viral drug which gained Gertrude Elion a Nobel Prize in 1988 (Elion, 1993), interferes with virally encoded thymidine kinase required for viral replication in non-dividing cells.

## Herpes Virus Life Cycle and the Intracellular Membrane Trafficking System

The process of viral entry, transport, and egress give clues as to whether a virus may be found in brain, and how Rab proteins may play a role in its replication cycle. HSV viral particles use cellular membrane trafficking to enter the cell, travel to the nucleus for DNA replication, and exit the cell through two membrane-envelopment steps, first between the nuclear membrane and then acquiring a second double membrane containing viral glycoproteins (Cheng et al., 2011), possibly via an autophagic-vacuole-type process (Ahmad et al., 2018).

HSV enters epithelial cells and neurons somewhat differently and may either fuse directly with the plasma membrane, liberating the viral capsid directly into the cytoplasm, or co-opt the endocytic pathway. When using the endocytic pathway, Rab GTPAses would be involved (Wandinger-Ness and Zerial, 2014).

In epithelial cells transport long distances is not as necessary as in the extended processes of sensory neurons (Penfold et al., 1994; Holland et al., 1999; Miranda-Saksena et al., 2000; Enquist et al., 2002; Douglas et al., 2004; Cunningham et al., 2006; Diefenbach et al., 2008; Diefenbach et al., 2016), while a number of other studies focus on the optic and trigeminal systems in mouse (Topp et al., 1994; LaVail et al., 1997; Garner and LaVail, 1999; Ohara et al., 2000; LaVail et al., 2007). Retrograde transport from synapse to cell body may rely on dynein (Topp et al., 1994; Sodeik et al., 1997; Dohner et al., 2002, 2006; Wolfstein et al., 2006), the retrograde microtubule-based motor that drives transport toward the minus end of microtubules, which primarily point toward the nucleus in axons (Vallee et al., 1988; Olenick and Holzbaur, 2019).

Previously, we demonstrated that NGF triggered retrograde transport of endosomes carrying phosphorylated Trk, the high affinity NGF receptor (Cui et al., 2007). In PC12 cells in culture, GFP-labeled NGF is endocytosed and moves in a stop-and-go manner from 0.93 to 2.29 μm/s (Cui et al., 2007), dependent on a functional dynein-microtubule network (Wu et al., 2007). Indeed, local modulation of plus-end microtubule motors targets the alpha-herpesvirus, pseudorabies, to either entry (retrograde) or egress (anterograde) (Smith et al., 2004). At later stages of epithelial cell infection, the microtubular system is disorganized, and uniform directionality of transport of viral particles to and from the nucleus is lost (Cheng et al., 2011) (and personal observations, Bearer, Ferland and Cheng).

We have used injection of particles into the isolated squid giant axon to study their transport capabilities. After injection into the giant axon, detergent-treated HSV-VP16-GFP traveled retrograde at an average instantaneous velocity of 2 μm/s (Bearer et al., 1999, 2000). Detergent treatment strips HSV of its envelope and other cellular membrane proteins rendering particles uniquely transported in the retrograde direction when injected into the giant axon (Bearer et al., 1999, 2000), suggesting that membrane-associate molecules are not needed for entry or transport to the nucleus. Since stripped virus lacks membranes, likely mediators of retrograde motor association must reside among the viral tegument proteins (Buch et al., 2017), an amorphous group some of which mediate capsid-envelope associations. Proteins removed by detergent, including APP, must therefore mediate anterograde transport of the virus (Satpute-Krishnan et al., 2003). APP is physically associated with HSV particles that travel in the anterograde direction in axons (Satpute-Krishnan et al., 2003), and a short 15 amino acid peptide derived from the cytoplasmic domain of APP can, on its own, mediate anterograde transport of inert plastic beads on microtubules in the giant axon via a kinesin-1 driven process (Satpute-Krishnan et al., 2006, 2012). In synchronized HSV-infected epithelial cells, viral particles labeled with VP26-GFP and membrane compartments containing APP-mRFP travel together during egress (Cheng et al., 2011). These compartments may include Rabs, although that has not yet been tested directly.

APP has a high affinity for kinesin-1, the conventional kinesin, and thus recruitment of cellular APP by a second, cellular transport-vesicle envelope displaying the cytoplasmic domain of APP toward the cytoplasm may explain HSV transport outbound during egress (Seamster et al., 2012) by kinesin-1 (DuRaine et al., 2018). Kinesin-3, another abundant microtubule-based motor in neurons (DeGiorgis et al., 2008), may collaborate (Kramer et al., 2012; Kratchmarov et al., 2013). Indeed, HSV particles likely recruit as many different types of motors and as many copies of each type as possible to mediate movements to and from the nucleus, independent of the orientation/polarity of microtubules, as we previously suggested (Cheng et al., 2011).

Here, we combine our technique for injection of defined particles into the intact giant axon with our understanding of transport in PC12 cells. Reconstitution of transport within a living axon of isolated particles allows precise characterization of the molecular composition of the particle, unlike other assays where the molecular composition of transported particles is a mystery. Injection of characterized particles into the axon also bypasses the main problem with extruded axoplasm, where precise buffer conditions if not correct can lead to false negatives.

**FIGURE 1:**
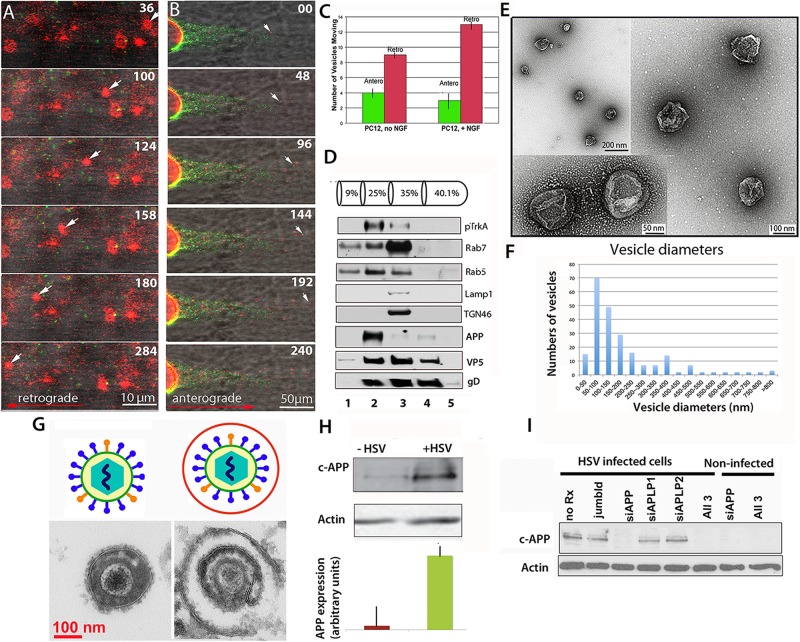
Axonal transport of endosomes isolated from PC12 cells in the squid giant axon and roles for APP and Rabs. **(A)** Membrane-bound vesicles purified from PC12 cells (Fraction 2) are transported retrograde after injection into a freshly dissected giant axon of the squid. Inert green fluorescent beads were co-injected. Most vesicles remain stationary, but one in this series (arrow) moves retrograde. Also see Supplementary Videos S1 and S2. **(B)** Vesicles (red), also move anterograde even when hCAPP peptide is co-injected., Peptide inhibits transport of beads (green) conjugated with hCAPP peptide, as previously described (Satpute-Krishnan et al., 2006). See Supplementary Video S3. **(C)** More vesicles move retrograde than anterograde and this difference increases after NGF treatment of the PC12 cells (*n* = 5, error bars = SEM). **(D–F)** Characterization of the vesicle preparation used in panels **(A–C)**. **(D)** Western blotting of fractions from PC12 cell organelle preparation showing co-fractionation at 25% sucrose of pTrkA, the high affinity NGF receptor, and Rab5 in fraction 2, the fraction that was injected in panels **(A,B)**. In contrast Rab7, LAMP1 and TGN46 concentrate in the slightly denser 35% fraction. Fraction numbers (1 ml) are indicated at the bottom. Fractionation of vesicles after radioactive NGF labeling showed that this Rab5B concentrates with I^125^-labeled NGF and phospho-TrkA in the 25% fraction (not shown). In parallel gradients of HSV-infected PC12 cells, APP also sediments in fraction 2 along with viral proteins, VP5 (capsid) and gD (envelope). **(E)** Examples of vesicles imaged by negative stain electron-microscopy demonstrate 50–300 nm diameter membrane-bound vesicles in fraction 2 from experiments shown in panels **(A–C)**. **(F)** Distribution of vesicle sizes in fraction 2. Three different fractionation experiments were examined by negative-stain electron-microscopy and vesicle sizes measured (*n* = 216). Average diameter across all three experiments was 180 nm, median size was 136 nm, and range was 42–1,200 nm. 75% of the vesicles fell between 50 and 300 nm. For the fraction injected in panel **(A)** (*n* = 42), the average size was 186, the median size was 140 nm, and range 10–500 nm. 80% of the vesicles fell between 50 and 300 nm (graph not shown). **(G)** Intracellular viral particles are found within a second membrane. Diagram (top). Thin section electron-microscopy (bottom) of extracellular (left) and intracellular (right) virions detects a second membrane surrounding enveloped particles within the cytoplasm of cells that is not present around extracellular virions. **(H)** HSV infection increases expression of APP *in vitro*. Western blots of PC12 cells with and without HSV infection demonstrated a large increase (25-fold) of APP as detected by the Zymed anti-C-APP antibody (Cheng et al., 2011). Shown is the 110 kD band representing full-length APP. Comparison of blot intensity by Image J (lower panel) quantifies the average change in arbitrary units. (Bars indicate range of triplicates.) **(I)** Suppression of APP by siRNA. APP expression in infected cells can be completely and specifically suppressed with siRNA in PC12 cells. In Vero cells, this suppression led to a dramatic decrease in production of infective virions (Cheng et al., 2011). *Methods:* PC12 cells were grown in a 15 cm dish plated at 2.3 × 10^7^ PC12 cells per plate (Wu et al., 2001; Cui et al., 2007). For labeling, cells were grown on coverslips and treated for 10 min with 5 μl of Vibrant DiI cell labeling solution (Molecular Probes/Thermo Fisher D3911) in 100 μl culture media. Cells were harvested and collected at 3k × *g*, resuspended in 0.5 ml 250 mM sucrose (28.6%), 10 mM Hepes, 1 mM imidazole, (pH 7.2), and debris pelleted at 800 × *g*. Supernatant was adjusted to 40% sucrose, put into a Beckman centrifuge tube and a sucrose gradient layered above (as shown in panel **D**) and spun at 100 × *g* in a TiSw501 Beckman rotor (Wu et al., 2001; Delcroix et al., 2003). Fractions were suctioned off, and sent frozen to Marine Biological Laboratory (MBL). Fraction 2 was co-injected with 1/100 dilution of deactivated beads (panel **A**) or C-APP conjugated beads (panel **B**). Green fluorescent beads (BioDesign) were prepared as described with or without peptide and de-activated in glycine (Satpute-Krishnan et al., 2006; Seamster et al., 2012). (Images captured with a 40× water immersion long-working distance lens on a 510 Zeiss scanning confocal microscope at 4s intervals for repeated sessions of 100 frames. PC12 cells were synchronously infected with HSV-GFP26 as described (Cheng et al., 2011) and vesicles (including intracellular viral particles) subjected to the same sucrose density gradient in parallel. For Western blots, fractions were TCA-precipitated, pelleted, resuspended in Laemmli buffer and loaded onto 10% SDS-PAGE. Blotting was performed as previously described (Wu et al., 2001; Delcroix et al., 2003; Wu et al., 2007; Xu et al., 2016). HSV blots were as described (Satpute-Krishnan et al., 2003). For electron-microscopy, 2 μl of vesicle fraction 2 was mounted onto formvar-coated, carbon shadowed de-ionized copper grids and negatively stained with 2 μl of filtered 1% aqueous uranyl acetate (EMSciences) as described for HSV (Bearer et al., 2000). Electron microscopy was performed on a Siemens CX200 electron microscope at MBL.)

We now report that endocytic vesicles purified based on their inclusion of the high-affinity NGF receptor, TrkA, are transported within the intact squid axon in both retrograde and anterograde directions in a fashion similar to envelope-stripped HSV capsid and intact virions (Figure 1). Endocytic vesicles from PC12 cells, a rat neural-type cell line, were labeled with DiI, a lipophyllic dye that labels membranes (Cheng et al., 2014), and isolated by flotation in a sucrose density gradient (see Figure 1 legend for “Methods”). In the axon vesicles moved retrograde as fast as 2 μm/s in a discontinuous fashion (Figure 1A), whereas HSV capsids also move at 2 μm/s retrograde but more continuously. These observations suggest that HSV capsid may have tighter motor binding (Satpute-Krishnan et al., 2006) since retrograde transport depends on only one type of motor, dynein. Thus differences in motility would not be due to motor-microtubule interactions, but rather to dynein’s relative affinity for cargo. While other studies have reported retrograde movement in extruded axoplasm (Pfister et al., 1989, Brady et al., 1990; Waterman-Storer et al., 1997, Sama et al., 2017), of all the cargo we have injected into the squid axon, only HSV capsids and TrkA containing vesicles move retrograde (Bearer et al., 1999, 2000; Satpute-Krishnan et al., 2003, 2006; Seamster et al., 2012). Thus capsid/tegument particles appear to recruit retrograde motors and their activators, including Rab GTPases, independent of membrane docking sites. PC12 vesicles also moved anterograde (Figure 1B). NGF treatment increased the proportion of retrograde moves (Figure 1C). Rab5 and APP were concentrated in the isolated fraction along with Trk (Figure 1D). By EM, 50% of the vesicles were 50–300 nm in diameter (Figures 1E,F), consistent with endosomal and transport organelles. Thin section EM detected secondary membranes surrounding intracellular virus (Figure 1G). HSV infection dramatically up regulated APP expression in PC12 cells (Figure 1H), which could be selectively suppressed by APP siRNA in PC12 cells (Figure 1I), as we have previously reported in epithelial cells (Cheng et al., 2011). It will be of interest to know if HSV infection up regulates APP expression in human brain.

A defect in axonal transport has been hypothesized as playing a role in AD (Stokin and Goldstein, 2006a, b; Brady and Morfini, 2017). Recent advances in imaging transport within the living brains of transgenic mice using manganese-enhanced magnetic resonance imaging (MEMRI) are elucidating relationships between AD, APP expression, presence of plaques and transport dynamics (Bearer et al., 2007; Simmons et al., 2008; Chuang et al., 2009; Gallagher et al., 2012; Majid et al., 2014; Medina et al., 2017).

## Rab Proteins in HSV Life Cycle

Rab GTPAses are a family of small GTPases that are widely distributed within the intracellular membrane systems, and are master regulators of trafficking pathways (Schwartz et al., 2007; Wandinger-Ness and Zerial, 2014; Zhen and Stenmark, 2015; Pfeffer, 2017). These GTP-hydrolyzing enzymes perform critical roles in all the intracellular membranes with which HSV interacts during its life cycle.

**FIGURE 2:**
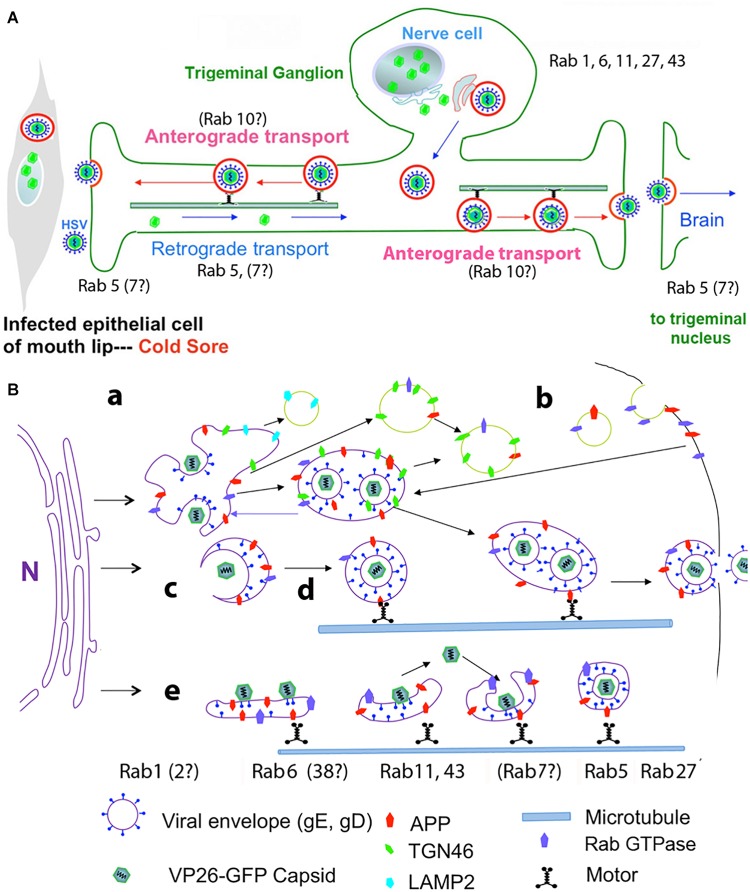
Diagrams of HSV intracellular dynamics. **(A)** Trigeminal ganglion neurons extend two processes – one outward to the mucous membranes of the lip, and the other inward to synapse in the trigeminal nucleus in the brain. Virus produced by the mucosal epithelial cells secondarily enters the sensory termini and travels retrograde to the neuronal nucleus where it may enter latency or be immediately replicated. DNA replication and capsid formation occur in the nucleus, secondary envelopment occurs in membrane systems within the cytoplasm, and motile virus travels out either process, to the lip or into the brain. Rab GTPases have been implicated in all these steps. **(B)** Cartoon showing viral interactions with intracellular membrane systems during egress in the cell body. This cartoon is based on observations in epithelial cells but a similar process is believed to occur in the neuronal cell body. Note that Rabs are also involved in the trafficking of APP. Colors and symbols representing various molecular and anatomical features are indicated at the bottom. Processes involving Rabs are indicated by letters: **(a)** nucleocapsids exit the nucleus; **(b)** recycling of viral glycoproteins to and from the plasma membrane may be involved in secondary envelopment; **(c)** nucleocapsids bud into Golgi-derived cellular transport vesicles; **(d)** enveloped virus inside a transport vesicle moves anterograde on microtubules; **(e)** capsids move in and out of cellular membranes during transit from Golgi to cell surface, or within axons to and from termini. Various Rabs are required for maintenance and dynamics of these cellular systems, but not all have been implicated in HSV egress (modified from Cheng et al., 2011).

The involvement of Rabs in HSV intracellular dynamics has been investigated primarily by knock-down or over-expression of Rab GTPase activating proteins (GAPs) experiments in HSV infected epithelial cells, accompanied by assessing infectious titers, viral protein expression, and imaging (either fluorescence- or electron-microscopy) (Figure 2).

In an initial experiment 37 different Rab GAPs were over-expressed in three types of human and mammalian epithelial cells (Zenner et al., 2011). Over-expression of partners for Rab1a/b and Rab43 were important for virion assembly. Depletion of these Rabs also resulted in lower viral production. In the absence of Rab1a/b, the viral glycoproteins were unable to traffic from endoplasmic reticulum, and thus un-enveloped particles built up in the cytoplasm. The defect resulting from Rab43 depletion was more complex, but apparently fragmentation and dispersal of the *trans*-Golgi network and its associated membranes rendered these compartments unable to support secondary envelopment of virions.

Other knock-down experiments demonstrated a role for Rab5 and 11 in viral entry into epithelial cells through the endocytic pathway (Hollinshead et al., 2012; Raza et al., 2018; Spearman, 2018). This study also suggested that these Rabs are involved in recycling viral glycoproteins from the plasma membrane to the *trans*-Golgi network where they may be recruited for secondary envelopment. Trafficking of viral glycoproteins from and to the plasma membrane may be a critical component by which virus escapes immune surveillance (Ndjamen et al., 2014).

Herpes simplex virus enters cells by at least two different processes: direct fusion between the viral envelope and the cellular plasma membrane; or endocytosis followed by fusion of viral and cellular membranes within the endosome. Viral glycoproteins left behind in the plasma membrane may be endocytosed independently for trafficking into the cell’s membrane-bound organelles and re-cycled to nascent particles. Thus whether either of these Rab GTPases, 5 and 11, are required for one or the other or both of these processes may be difficult to dissect without more precise tools.

Knock-downs of other Rabs demonstrated roles for Rab6, 27 and 43 in HSV life cycle. Rab6 appears mainly in the Golgi and cytoplasmic vesicles and appears to be involved in transport between ER and Golgi and may also participate in plasma-membrane to Golgi trafficking of membrane compartments (Figure 2B). Proteomics by mass spectroscopy of intracellular HSV particles detected Rab 4, 5, and 7 (Arkady Rasin and Elaine Bearer, personal communication). Microtubule-based motors, dynein and kinesins, have been identified as possible effector proteins for Rab6. Knock-down by siRNA of 60 different Rab proteins identified Rab6 as the principle Rab involved in virus production (Johns et al., 2014). Knock-down of Rab6 blocks Golgi-to-plasma membrane transport of virions by a pathway used by several integral membrane proteins but not by luminal secretory proteins. Rab6-dependent viral packaging was facilitated by ERC1, a Rab6-interacting protein linking microtubules to the plasma membrane (Monier et al., 2002). ERC1 is an ELKS/RAB6-interacting/CAST family member, where ELKS appears to route Rab6 of the secretory pathway to lysosome-type organelles in cultured melanosomes (Patwardhan et al., 2017).

Rab27 is involved in exocytosis and supports surface transport of envelope proteins of other enveloped viruses, such as the parainfluenza family that bud from the plasma membrane (Ohta et al., 2018). A role for Rab27 in HSV life cycle has been proposed based on co-localization with tegument (GFP-UL46), although not with capsid (GFP-UL35) in oligodendrocytes (Bello-Morales et al., 2012). Depletion of Rab27a produced a significant decrease in numbers of infected cells and viral production. Ultimately extracellular mature infectious virus is primarily enveloped in its glycoprotein-rich lipid bilayer. However, extracellular viral particles may also only be enveloped with cellular-derived membranes (exosome-like), which would allow the particles to escape immune system surveillance (Bello-Morales and Lopez-Guerrero, 2018). How such particles, lacking viral glycoproteins, would enter cells and through what compartment remains to be discovered.

## Rabs and APP

Rabs interact with the same membrane compartments as APP and HSV (Xu et al., 2016). Full length APP or its proteolytic C-terminal fragment(s) such as the beta cleavage product beta-CTF have been shown to reside in Rab5-positive endosomes in axons. Excessive APP or beta-CTF impaired axonal movement of Rab5 endosomes (Weissmiller et al., 2015; Xu et al., 2016). Deregulation of Rab5 activity and trafficking is one of the early cellular pathologies in AD that precedes the appearance of amyloid plaques and neurofibrillary tangles (Cataldo et al., 2000; Stokin et al., 2005; Xu et al., 2018; Xu and Wu, 2018).

## Rabs as Targets for Drug Therapy of Herpes-Associated Cognitive Decline

Intracellular membrane systems interact dynamically on very short (ms) time scales. Disruption of Rabs perturbs the delicate balance of these many moving, fluid compartments. Hence it is no surprise that knockdowns of the management system, the Rabs, would interfere with packaging of enveloped virus, and all other steps in the viral infection and production process. Whether this interference is direct, through interactions with the viral products, or an indirect consequence of cellular membrane trafficking disruption, remains unclear. More precise interventions will be needed to identify a specific role for any of the Rabs in HSV life cycle. Tools for such specific interference could include modified effector molecules that bind to viral proteins and inhibit or promote Rab binding to membranes containing viral products, or drugs that interfere with membrane lipid modifications required for Rab membrane docking. Such experimental manipulations could also have a therapeutic use.

Are the Rab GTPases potential therapeutic targets for HSV infections? If, as we suggest here, HSV re-activation is a risk factor for brain pathology such as (but not limited to) AD, then could inhibition of a specific Rab GTPase be a potential therapy? This idea has been proposed previously (Stein et al., 2003; Raza et al., 2018). HSV is not the only pathogen to recruit Rabs. Most enveloped viruses are likely to interact with intracellular membrane compartments and encounter Rabs. Examples of this are parainfluenzavirus (Ohta et al., 2018), cytomegalovirus (Homman-Loudiyi et al., 2003; Krzyzaniak et al., 2009; Fraile-Ramos et al., 2010; Indran and Britt, 2011; Lucin et al., 2018), and HIV (Murray et al., 2005; Chu et al., 2009; Qi et al., 2013, 2015; Ahmad et al., 2018). Intracellular bacteria may also use Rabs (Stein et al., 2012; Spano and Galan, 2018).

Rabs have also been implicated in cancer progression, with the consequence that targeted Rab inhibitors are under development (Salehi et al., 2009; Hong et al., 2010; Agola et al., 2015; Raza et al., 2018). The ubiquitous activity of Rabs in all aspect of intracellular membrane dynamics requires that any such pharmaceutical intervention be tightly directed to the pathologic process. Side effects and off-target effects could be serious challenges. However, a specific inhibitor of another GTPase, Rac1, is under development for ovarian cancer treatment and seems promising in cases where the Rac1 is abnormal (Hudson et al., 2018). Such drugs could ultimately become another general or specific anti-viral and anti-intracellular bacterial treatment, an area of pharmaceuticals in need of new ideas.

## Data Availability

All datasets generated for this study are included in the manuscript and/or the Supplementary Files.

## Author Contributions

EB developed the ideas, directed the experiments and wrote the manuscript. CW provided the labeled endosomes, added insight to the ideas, performed some of the Western blots, participated in the experiments and interpretation, edited the manuscript.

## Conflict of Interest Statement

The authors declare that the research was conducted in the absence of any commercial or financial relationships that could be construed as a potential conflict of interest.

## Footnotes

1 https://www.nia.nih.gov/alzheimers/clinical-trials/valacyclovir-mild-alzheimers-disease
