# Supplementary material for: Herpes Simplex Virus, Alzheimer’s Disease and a Possible Role for Rab GTPases
Source: Front Cell Dev Biol. 2019 Aug 7;7:134. doi: 10.3389/fcell.2019.00134 (PMC6692634; doi:10.3389/fcell.2019.00134)
Supplement: Supplementary file 4 [file Data_Sheet_1.PDF]

Bearer: HSV, Alzheimer's disease and Rabs

Supplemental Materials

Supplemental Video S1: A giant squid axon injected with labeled endosomes shown in Fig. 1 together with green fluorescent beads (de-activated). Imaging was on the retrograde (towards the axonal nuclei) side of the injection site. Endosomes (red) are mostly stationary, but two move retrograde in a stop-and-go fashion at about a 2µm/sec instantaneous velocity. Beads were treated and imaging was performed as we described (Satpute-Krishnan et al, 2006 PNAS).

Supplemental Video S2: Cropped video from the one shown in S1 focusing on the movement of one retrograde endosome, the same indicated with an arrow in static frames in Figure 1A.

Supplemental Video S3: A giant squid axon injected with PC12 endosomes. Video of the same sequence as still frames shown in Fig. 1B and imaged on the anterograde side (towards the synapse) of the injection site, which appears as a large sphere at the left of the frame. Green hCAPP beads (1:10; BioDesign) mixed with PC12 fraction 2 labeled with C99-mRFP and hCAPP peptide (2.5mg/ml) were co-injected as described in Satpute-Krishnan et al 2006 and Seamster et al 2012. Note that the green beads are mostly stationary and many red vesicles move anterograde. This suggests that soluble peptide can inhibit peptide-conjugated beads but not endosomes displaying the full length C-terminus together with other potential motor recruitment molecules, such as JIP1. Peptide also does not inhibit positively charged beads from being carried anterograde, presumable because motors don't bind specifically to positive charge (not shown).
